# Supplementary material for: Differential Methylation Profile in Fragile X Syndrome-Prone Offspring Mice after in Utero Exposure to Lactobacillus Reuteri
Source: Genes (Basel). 2022 Jul 22;13(8):1300. doi: 10.3390/genes13081300 (PMC9331364; doi:10.3390/genes13081300)
Supplement: Supplementary file 1 [file genes-13-01300-s001.zip › genes-1800462-supplementary.pdf]

SUPPLEMENTARY INFORMATION

Supplementary Figures

Figure S1. Overlap of CpGs passed quality control (PassedQC) and with unique best alignment score (UniqueAlign). Only the CpGs with unique best alignment score and passed quality control are used in the analysis to find different methylated CpGs.

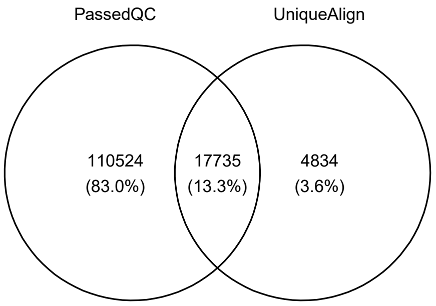

Figure S2. β value distribution of (A) all CpGs passed quality control and (B) CpGs passed quality control and with unique best alignment score.

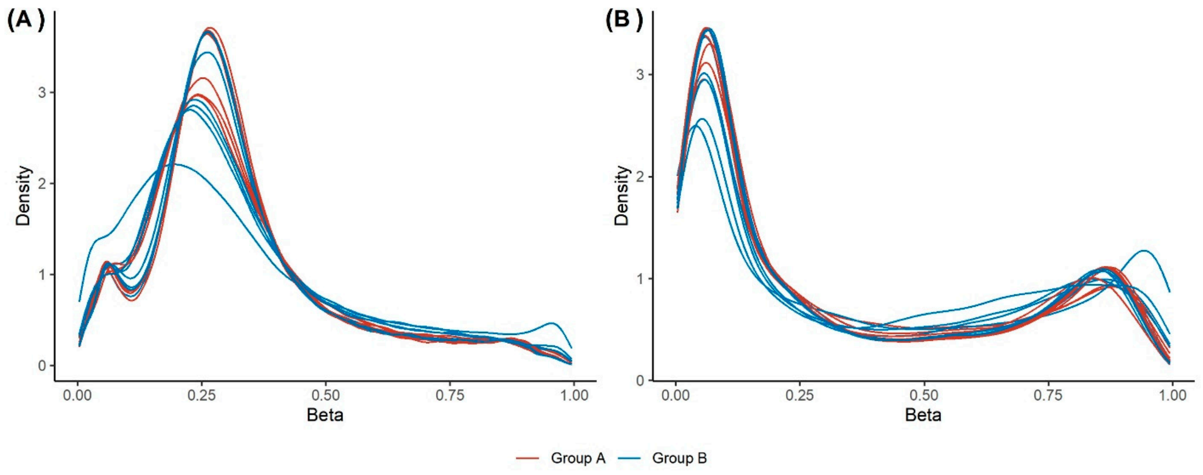

Supplementary Tables

Supplementary Table S1. List of differentially methylated CpG sites and their corresponding genes.

probeID	chr	geneSymbol1500	logFC	*P*.Value
cg04523291	6	Oxtr	0.184336	0.484761
cg15317815	6	Oxtr	-0.3704	0.623746
cg02192228	6	Oxtr	-0.27426	0.672402
cg02313829	7	Klhl35	1.020785	0.002424
cg04830124	4	Casz1	-0.96639	0.003318
cg16113298	2	Pax6	2.432251	0.004143
cg16776421	17	Rab11fip3	3.457276	0.004376
cg18861001	8	Zfp423	0.577008	0.004565
cg09125011	2	Barhl1	-0.83119	0.00462
cg12804010	3	Syt6	-1.96937	0.006188
cg19175742	7	Ap3b2	-0.68573	0.006662
cg02181847	13	Tfap2a	2.897984	0.007404
cg12827760	1		-0.62317	0.007493
cg08102602	6	Ptms	2.306798	0.007932
cg04665204	11	Bahcc1	1.321405	0.008311
cg04978747	5	Agap3	0.729015	0.008324
cg19382919	2	Sp5	2.237178	0.008451
cg03319479	2		2.603797	0.009032
cg21739318	8	Klhdc4;Galnt2l	2.373626	0.009155
cg17723677	12	Ankrd9	0.878877	0.009182
cg10187792	11	Rab37	2.624925	0.009272
cg17553854	5	Epha5	-0.65265	0.009963
cg13874817	9	Spsb4	-1.15326	0.009969
cg06839483	4	Myorg	2.702576	0.010356
cg26529376	2	Kcnip3	-1.35701	0.010897
cg23646337	7	Tmem145	-1.00392	0.010951
cg07206010	X	Trex2	-0.64557	0.01113
cg01824138	4	Casz1	2.492807	0.011195
cg11937129	5	Tmem120b	2.601914	0.011328
cg26287163	13		2.041793	0.011419
ch.15.1310449F	9	Morf4l1	2.272031	0.011721
cg14166458	4	Mrto4;Ubr4;Emc1	0.578276	0.011767
cg01808706	2	Itpka	-0.76641	0.011965
cg00420869	11	Adra1b	1.534408	0.012092
cg11482571	2	Tfap2c	-0.74666	0.012402
cg13456504	5		-0.52014	0.012699

cg08651034	17	Tmem200c	3.051603	0.012968
cg04072768	11	Sptbn1	-0.67996	0.01303
cg03380643	2	Insm1	-1.63372	0.013074
cg09537448	6		3.56636	0.013169
cg14664922	7	Usp47	-0.56938	0.0132
cg15755084	5	Msx1	2.231316	0.013279
cg19370097	16	Zbtb20	3.317076	0.013326
cg17811167	11	Mllt6	-0.67076	0.013446
cg25536300	10	Ptbp1	-0.51927	0.013566
cg11078828	7	Clpb	2.273617	0.014196
cg05565239	12	Rtn1	2.38777	0.014337
cg18567174	9	Skor1	-1.40252	0.01441
cg05770797	11	Nr1d1	-0.85059	0.014468
cg07204662	19	Esrra	2.743779	0.014506
cg22652733	10	Mcu	2.69605	0.014538
cg24775323	9		-2.62253	0.014543
cg02063736	2	Rbm39	2.194327	0.014561
cg21321248	5	Kcnh2	2.665116	0.014691
cg16069003	3	Rsrc1	-0.83167	0.014712
cg15827532	5	Lrch4;Gm20605	-0.77403	0.014823
cg27598576	15	Atf7;Gm28047	2.197171	0.014941
cg02474076	6	Gcc1	1.832169	0.014957
cg13090987	7	Slco3a1	-0.55961	0.015011
cg14769525	2		-0.66983	0.015028
cg19504123	11	Rab34	-0.58855	0.015178
cg02368058	5		-0.85374	0.015206
cg02453348	7	Xntrpc;Trpc2;Xndc1	-0.62145	0.015269
cg22617643	1	Rpl7;Rdh10	1.828795	0.015278
cg07298473	2	Nr1h3	1.509187	0.015588
cg01501208	7	Nr2f2	2.294977	0.015598
cg10614025	7	Ap3b2	-0.75483	0.015638
cg00795277	11	Fgf11	2.331615	0.015782
cg05486213	2	Mpped2	1.759267	0.015818
cg16468913	X	Cd40lg	-0.56999	0.01601
cg09157431	11	Pafah1b1	2.665805	0.016204
cg23114866	2	Nkx2-2	2.701786	0.016272
cg23333820	19	Psd	2.107466	0.016469
cg02296807	9	Atg4d	0.671568	0.016516
cg19425477	9	Lingo1	-0.59893	0.016531
cg09675845	7	Dlg2	2.839827	0.016552

cg19802138	8	Sox1	2.192066	0.016762
cg13239348	7		-0.76917	0.016808
cg01311718	1	Ikzf2	2.30294	0.01681
cg10170306	2	Arpc5l	3.069299	0.016869
cg16734637	6	Foxp1	-0.64361	0.016892
cg14421309	2	Zeb2	2.236837	0.016901
cg06295260	2	Duox1	1.946971	0.017016
cg20332660	4		2.015952	0.017503
cg03620623	7	Ap2a1	-0.71569	0.017813
cg07249939	17	C2;Zbtb12	-0.58475	0.017939
cg21501358	18	Klhl14	1.766403	0.018023
cg19853848	18	Sting1	0.7154	0.01804
cg01181227	5	Nkx1-1	2.159131	0.018086
cg17520144	11	Dhrs11	2.526551	0.018143
cg24002886	10	Col6a1	1.616632	0.018324
cg07060175	11	Tenm2	2.200879	0.018332
cg05007549	5	Prdm8	2.82459	0.018558
cg03669936	17	Fbxo11	-0.66907	0.018693
cg22373415	9	Smad6	-0.49945	0.01902
cg22424903	17	Tead3	2.382026	0.019099
cg06887471	6	Zfp777	-1.14782	0.019156
cg08296191	15	Shank3	1.642875	0.019285
cg16194588	7	Lmtk3	-0.83297	0.019347
cg03743119	9	Smad6	-0.84152	0.019556
cg10433594	10	Cited2	2.050123	0.019614
cg19619003	4	Foxo6	2.258653	0.019767
cg02756545	3	Abcd3	-0.61713	0.019818
cg16642517	5	Rbm47	-0.60463	0.020173
cg15172734	14	Slmap	0.895535	0.020331
cg03536289	12	Asb2	-0.64765	0.020352
cg26780404	9	Col12a1	1.982699	0.020414
cg20390613	4	Dhrs3	-0.99101	0.020456
cg18267374	14	Nefm	1.535546	0.02053
cg26073720	5		1.968082	0.020686
cg03122669	15	Hoxc9;Hoxc5	-0.48616	0.020791
cg16984132	9	Sidt2	-1.12145	0.02093
cg05493495	9	Peak1;Hmg20a	-0.65556	0.021144
cg07819065	11	Septin4	2.27893	0.021195
cg07342016	X	Rab33a	2.08166	0.021407
cg04431387	15	Phf5a;Aco2	1.536859	0.021553
cg13770291	13		2.19203	0.021615

cg15416233	19	Pax2	2.057116	0.021619
cg06225294	16	Zbtb20	2.670956	0.021754
cg06566772	10	Arid5b	-0.87675	0.021804
cg15663265	8	Dnase2a;Mast1	2.803789	0.02189
cg24156613	X	Bcor	-0.66785	0.02192
cg21285133	1		2.025302	0.022039
cg07035165	18	Kcnn2	2.850647	0.022118
cg19084362	3	Insrr;Pear1	2.558831	0.022178
cg10573553	10	Dip2a	-1.83138	0.022206
cg04289959	7	Fbrs	-2.02808	0.022248
cg16944597	16	Fam131a	-0.51397	0.022269
cg16464569	4	Fhl3	2.196882	0.022409
cg01651290	7	Dusp8	-0.8178	0.022469
cg03437025	15	Sp1	1.66144	0.022546
cg27400113	11	Meis1	1.69064	0.022674
cg00147172	15		-0.62588	0.022753
cg07014673	1		2.199832	0.022791
cg11881754	5	Srrm3	-2.09956	0.023028
cg16039964	11	Rnasek;0610010K14Rik;Gm21988	-0.72949	0.02305
cg13352750	6	Hoxa11	-1.98004	0.023074
cg01469884	6	Tril	1.824439	0.023096
cg19186380	15	Hoxc5	2.91683	0.023385
cg14848832	3	Efna3	1.079247	0.023434
cg10649367	18	Zfp521	1.510501	0.023571
cg08029967	2	Olfr1195	1.335881	0.023655
cg06392318	10	Rspo3	2.086661	0.023791
cg04419618	X	Rbmx	1.598277	0.023807
cg16423747	11	Nf2	-0.56957	0.023867
cg03272508	19	Sf1	1.772008	0.023885
cg12616720	X	Nkrf	1.977865	0.023937
cg06312785	9	Ppp2r3a	-0.53217	0.023978
cg03341655	8	Gfod2	0.719992	0.024007
cg24636368	11	Skap1	1.97862	0.024099
cg02651788	X	Arx	1.740141	0.02433
cg17933765	1	Rabif	-0.70964	0.02434
cg06626750	11	Srsf1	2.135296	0.024373
cg06082962	19		2.884829	0.024537
cg10441013	1	Kcnj10	2.334909	0.024633
cg22904096	1	Myl1	-0.5163	0.024716
cg01640150	1	Vcpip1	2.265291	0.024822

cg05823643	11	Dynll2	1.535811	0.024908
cg22821358	1		-0.63558	0.025048
cg04653627	17	Col11a2	-0.56186	0.025142
cg24626752	14	Slitrk5	-0.55847	0.02544
cg20381092	11	Sp6	1.855437	0.025482
cg25353120	19	Arl3	-0.43884	0.025482
cg17009731	17	Prrc2a	1.99474	0.025621
cg14473568	16	Robo2	1.685054	0.025634
cg25987923	19	Emx2	-0.59499	0.025636
cg14322166	X	Nlgn3	1.391869	0.025738
cg08779207	2	Plcb2	0.778785	0.025895
cg10254000	16	Tbx1	2.459305	0.02604
cg05067991	5	Kdm2b	1.137273	0.026048
cg05942831	8	Zfp703	-0.57884	0.026187
cg25520488	11	Tanc2	-0.52405	0.026256
cg26688315	1		-0.86551	0.026274
cg06759265	4	Dlgap3	-2.62187	0.026313
cg04616652	1	Hmcn1	2.158481	0.02644
cg02187816	3	Elf2	2.31534	0.026447
cg11097249	11	Tenm2	-1.07153	0.026531
cg19781843	4	Ncdn	0.432567	0.026584
cg21743182	5	Cdk14	2.932356	0.026873
cg24027179	11	Myh10	-0.67677	0.027022
cg08393828	12	Ppp2r5c	-0.58697	0.027081
cg05469614	17	Zscan10	1.642401	0.027253
cg02856606	4	Iffo2	1.458523	0.027255
cg08948849	9	Ankdd1a	1.876079	0.027354
cg03035653	X	Pcdh19	-1.21918	0.027428
cg13886231	15	Gga1	0.583402	0.027539
cg27647370	15	Tcf20	2.163175	0.0276
cg10363915	2	Zdhhc5	0.797301	0.027622
cg15554875	17	Six2	-0.85318	0.027675
cg13248877	16	Tnk2	1.728833	0.027688
cg06767010	9	Spsb4	2.567862	0.027713
cg11316904	6		-0.75723	0.027796
cg06206603	14	Sec24c	1.7641	0.027859
cg07069878	9	Stag1	2.131334	0.027905
cg15279476	10	A930033H14Rik;Rhobtb1	0.663279	0.028054
cg25734680	13	Zmynd11	-1.36966	0.028151
cg06910048	9	Pih1d2;Nkapd1	2.209947	0.028295

cg02153528	10	Ankrd52;Nabp2	-0.69989	0.028302
cg02511809	18	Apc	-1.16468	0.028457
cg00399596	6		-1.30062	0.028467
cg12045059	9	Nlrx1	1.833991	0.028672
cg14785750	1	St18	1.847807	0.028725
cg05223760	X	Foxp3;Ppp1r3f	-0.42098	0.028798
cg06053959	11	Tlcd2	-0.68596	0.028992
cg01655356	7	Shank2	-0.6903	0.028996
cg22441074	11	Mnt	1.708174	0.029066
cg00314427	7	Fosb	2.425453	0.029162
cg21027282	12	Rad51b	-0.79248	0.029277
cg24727620	5	Tmem243;Dmtf1	-0.63932	0.029359
cg12815918	5	Pdx1	1.919855	0.029397
cg11381282	14	Gjb2	1.226515	0.029423
cg01391538	4	Trnp1	-0.45672	0.029475
cg08554115	11	6330403K07Rik	-0.53656	0.029608
cg17451978	9	Ccdc13	-1.4302	0.029701
cg20460154	3	Alpk1	1.612764	0.029749
cg01204606	9		-0.62511	0.029908
cg13045637	12	Prkch	1.898537	0.029929
cg17553300	14	Wnt5a	-0.48268	0.029954
cg00494665	10	Tbc1d30	1.969182	0.030016
cg17026973	11	Wdpcp	0.682991	0.030018
cg01789705	7	Gtf3c1	1.986237	0.030079
cg11700230	17	Col11a2	2.609239	0.030104
cg15713282	9		1.541953	0.030145
cg04203883	15	Hoxc11	1.685362	0.030216
cg10251328	11	Igf2bp1	1.975449	0.030398
cg25517592	8	Zfp827	1.506392	0.03041
cg10882692	4	Kif1b	2.302598	0.030428
cg22138822	5	Pole	2.163065	0.030512
cg01743962	11	Rai1	2.296861	0.030539
cg19974474	10	Gdf11	-0.87172	0.030573
cg10428279	2	Gabpb1	2.35727	0.030695
cg16001135	5	Adap1	-1.12402	0.030905
cg11164537	11	Zkscan17	-2.19332	0.031025
cg24142850	8		2.030971	0.031177
cg23671921	11	Ccdc88a	-0.84747	0.031371
cg06410537	11	Tlx3	-0.82893	0.03148
cg17140476	2	Ccdc32	1.434967	0.031526
cg14568422	18	Tcf4	-0.56162	0.031543

cg15597945	6	Lrp6	-0.53101	0.031653
cg09703759	2	Nsmf	-0.54674	0.031658
cg16764637	6	Gm28308;Hoxa6;Hoxa3	1.817616	0.031809
cg03877957	11		2.402941	0.031877
cg10229294	5	Lhx5	1.878838	0.031896
cg18200760	3	Ntng1	1.841689	0.031922
cg05811612	7	Bicra	1.949487	0.031946
cg20654074	2	Dll4	1.636187	0.031983
cg25019126	3	Elovl6	1.910309	0.031993
cg23774780	1	Cdc42bpa	1.910866	0.032138
cg04580151	4	Faah	0.821237	0.032428
cg10471976	11	Rundc3a	2.530395	0.032466
cg15477011	6	Gm20696;Foxp1	-0.72633	0.032554
cg03234775	17	Agpat1	2.261016	0.032577
cg09595163	14	Wnt5a	-0.70146	0.032611
cg12713525	2	Mpped2	1.924898	0.032611
cg14841796	17	Gabbr1	2.261918	0.032623
cg26878182	2		1.386508	0.032624
cg10030162	8	Zfp423	1.973902	0.032675
cg13067005	2	Ube2e3	-1.23184	0.032703
cg20403876	6	Dctn1	2.019048	0.032747
cg12537546	11	Rnasek;0610010K14Rik;Gm21988	1.954066	0.032759
cg16409838	15	Septin3	-0.89042	0.032795
cg16262623	11	Ubtf	1.956235	0.032824
cg24806294	15	Scrt1	1.954433	0.032907
cg12688234	11	Insyn2b;Dock2	0.47316	0.032947
cg24024424	18	Pcdhgb7;Gm42416;Pcdhgc3;Pcdhga5;Gm37388;Pcdhga11;Pcdhga6;Pcdhgb4;Pcdhga12;Pcdhgb6;Gm37013;Pcdhga3;Pcdhga7;Pcdhga4;Pcdhga9;Pcdhga1;Pcdhga8;Pcdhga10;Pcdhgb1;Pcdhga2;Pcdhgb5;Pcdhgb2	2.396577	0.033041
cg02577985	5		-0.62032	0.033231

cg15259986	2	Fign	1.840343	0.033252
cg22402261	4	Ncdn	2.036832	0.03339
cg04212619	6	Slc6a6	1.708033	0.033426
cg14542590	12	Foxn3	1.764257	0.033535
cg18849004	2	Fmn1	-0.95307	0.033543
cg04402966	5	Dbf4;Slc25a40	-0.56425	0.033549
cg26820000	4	Nfyc	-0.54098	0.03359
cg24678862	13	Irx1	1.007134	0.0336
cg05090186	1	Zbtb18	-0.54969	0.033662
cg02913140	13	Mef2c	2.189952	0.03403
cg09093100	4	Rnf220	-0.4934	0.034075
cg00513735	13	Hk3;Unc5a	0.3956	0.034124
cg19081437	11	Hoxb4	1.810101	0.034138
cg16178942	8	Arhgef7	1.975601	0.034188
cg07819688	5	Fosl2	-1.59106	0.034196
cg26921093	11	Axin2	2.337295	0.034224
cg05949352	2	Prdm11	-0.96624	0.034264
cg00871390	5	Atg9b	2.246687	0.034267
cg00445443	6	Hoxa2;Gm28308	1.567558	0.034328
cg20392607	6	Kcna1	1.725726	0.034349
cg14764542	5	Ppp1cb	-0.47103	0.034427
cg04875128	7	Otud7a	-0.50466	0.034473
cg22286687	11	Hexim2	0.703714	0.034483
cg06835212	13	Mef2c	-0.56536	0.034525
cg04941520	8	Atxn1l	2.452002	0.03457
cg21418679	1	Fbxo36;Trip12	-0.92377	0.034637
cg17599620	9	Clasp2	2.480497	0.034686
cg04074140	9	Col12a1	1.619798	0.034777
cg26238975	12		-2.45071	0.034794
cg07680319	2		1.458069	0.034982
cg21386952	1	Col6a3	-0.56457	0.034997
cg24030086	5	Kmt5a	1.741675	0.035016
cg16032050	14	Prrxl1	0.566578	0.03509
cg10448436	2	Pax6	2.454045	0.035192
cg20418711	16	Runx1	-0.48492	0.035524
cg03982541	12	Brf1	1.967336	0.035546
cg15929451	6	Foxp1	1.329787	0.035575
cg19338524	5	Nkx1-1	2.391119	0.035602
cg06414929	2		1.742138	0.035628
cg20908614	9	Atp2c1	2.155909	0.035702
cg03697509	7	Stard10	1.896703	0.035935

cg09910967	10	Heca	-0.50893	0.035978
cg16872560	6	Ppp1r9a	1.870555	0.036152
cg23398076	11	Meis1	1.898709	0.036269
cg11589723	7	Bicra	1.873304	0.036295
cg06822120	5	Nat8l	1.915494	0.0364
cg15797110	6	Hoxa7	1.752735	0.036424
cg00313219	19	9930021J03Rik	1.760916	0.036538
cg03304815	9	Amotl2	1.392802	0.036579
cg14733594	15	Dab2	1.584526	0.036596
cg14948714	11	Patz1	-0.56682	0.03669
cg26813561	5	Acad10	2.175321	0.036772
cg14086160	1	Igsf9;Tagln2	-0.74014	0.036797
cg05913638	9	Dscaml1	-0.96001	0.036843
cg14665326	10	Plxnc1	1.979496	0.036918
cg07879727	11	Wnt3	1.731227	0.037039
cg04637372	4	Vwa5b1	-0.49079	0.037045
cg07906484	2	Hnf4a	1.65938	0.037217
cg27359731	7	Erfl	-0.48033	0.037241
cg12492753	15	Kmt2d	1.862386	0.037263
cg19697359	4	Epha8	1.755918	0.037297
cg17536413	4	Hectd3	-0.88023	0.037312
cg20059876	4	Grik3	-1.47202	0.037449
cg21177673	4	Dab1	1.918809	0.03756
cg00575115	2	Pcif1;Zfp335	2.154654	0.037585
cg20184165	3	Naa15	1.918196	0.03766
cg16787065	19	Ovol1	2.422299	0.037739
cg26884359	11	Zfp385c	1.430583	0.037748
cg25797455	5	Tsc22d4	-2.07189	0.037772
cg09940360	17	Ttbk1	-0.51169	0.037779
cg05835276	10	Nxph4	2.51479	0.037782
cg24791025	3		-0.40767	0.037793
cg27458485	10	Anks1b	2.296618	0.037838
cg16383389	3	Bcar3	-0.63336	0.037852
cg02171705	X		1.586473	0.037861
cg17047659	6	Hoxa10;Hoxa11	1.935169	0.037974
cg20065832	X	Pou3f4	-0.73969	0.038005
cg12001120	1	Wnt10a	0.597434	0.038152
cg14385804	1	Mff	2.445702	0.038175
cg06063729	9	Rassf1	1.867808	0.038181
cg06541294	9	Msl2	1.664525	0.038246
cg14846463	6	Wnt7a	-0.58431	0.038315

cg19080590	8	Cdh13	-0.46512	0.038364
cg15815114	X	Cul4b	-0.63203	0.038389
cg02119494	9		1.863657	0.038531
cg03256465	4	Jun	-0.62068	0.038532
cg20511797	2	Abl1	1.709022	0.038554
cg21448057	14	Slitrk1	-0.61341	0.038709
cg07601212	12		2.339174	0.03885
cg22310240	13	H2ac12;H2bc12	1.733738	0.038915
cg14433153	13		1.889507	0.039001
cg11614389	13	Ssbp2	1.674648	0.039021
cg01305421	10	Igf1	-0.87565	0.039034
cg20961037	11		1.646058	0.039046
cg10381455	15	Fignl2	-0.52515	0.039079
cg17511933	1	Gli2	-0.53178	0.039124
cg12244464	15	Gga1	-0.45221	0.039125
cg18305324	17	Prrc2a	1.628956	0.039145
cg04813639	9	Rasgrf1	2.050192	0.039155
cg04868764	2	Spint1	-0.48578	0.039366
cg12072393	16	Dvl3	0.665338	0.039388
cg24118894	7		2.080453	0.039397
cg09400281	12	Eml1	-0.48859	0.039463
cg23150604	6		1.98671	0.039483
cg05146658	1	Agap1	2.60674	0.039489
cg22120094	15	Zhx2	0.951318	0.039611
cg04557908	4		1.889449	0.039646
cg25553110	15	Zhx2	-1.11794	0.039669
cg18355562	13		-0.65879	0.039685
cg08933237	6	Magi1	-0.42995	0.039724
cg08540100	4		1.990581	0.039726
cg00713286	11	Rap1gap2	-0.49287	0.03984
cg26168324	19	Emx2	2.368946	0.039922
cg24032214	7	Leng1;Cnot3	-2.50475	0.039959
cg05059566	5	Prdm8	1.444865	0.040029
cg01951459	1	Agap1	-1.97016	0.040098
cg13328485	2	Ubr3	0.546452	0.04015
cg11688073	8	Tent4b	1.768226	0.040187
ch.20.829586R	2		2.098125	0.040228
cg07851057	13	Bdp1	-0.63187	0.040632
cg17844121	6	Ppp1r3a	1.933004	0.040652
cg08900073	9	Bcl9l	-0.76757	0.040686
cg15336196	18	Setbp1	1.43452	0.04073

cg01506917	17		2.150766	0.040731
cg06398166	9	Gmppb;Ip6k1	-1.04919	0.040764
cg09894929	6	Nxph1	-0.85616	0.040803
cg14996807	9	Unc13c	-1.63113	0.0409
cg20786058	13	Fst	1.930786	0.040924
cg17124583	2	Gata3	-0.63451	0.04093
cg00109797	8	Helt	-1.4113	0.040986
cg11067405	12		2.174638	0.04099
cg03586128	7	Dock1	-0.66373	0.041019
cg07790870	2	Cbfa2t2	1.418216	0.041074
cg21059834	5		0.484826	0.041082
cg24599205	3	Kcnc4	-0.43274	0.041141
cg11556846	1		1.471209	0.0412
cg19317565	10	Syn3	1.136407	0.041276
cg12690246	15	Aqp5	-0.68011	0.041305
cg22336337	1	St18	1.84722	0.041507
cg01443426	15	H1f0	1.591349	0.041527
cg02991558	9		2.131406	0.041637
cg14175580	1	Camsap2	2.273818	0.041765
cg24352736	9	Timm29;Yipf2;Carm1	1.652526	0.041823
cg26413942	18	Zfp608	1.727224	0.041921
cg24372550	1	Epha4	1.863367	0.041929
cg19210770	1	Asic4	-1.45023	0.041932
cg04068518	12		2.172538	0.041952
cg09604618	2	Mpped2	2.118858	0.042041
cg24005685	3	Pitx2	1.894964	0.042128
cg02951021	9		1.818004	0.042241
cg21573538	2	Gchfr	-0.5314	0.042253
cg08504765	8		1.666345	0.042316
cg03181473	4	Trabd2b	1.382602	0.042367
cg15648389	15	Hoxc4	1.785791	0.042398
cg25266232	18	Dcc	2.194403	0.042414
cg10752869	2	Meis2	0.536204	0.042424
cg15412759	1	Tfap2d	1.997425	0.042446
cg11171004	1		1.582684	0.042525
cg15966253	14	Ube2e1	-0.45379	0.042593
cg09895223	X	Chrdl1	-0.82057	0.042672
cg11413800	8	Mast1	1.324687	0.042676
cg06990796	11	Wwc1	-0.50218	0.042707
cg19302474	4		1.369852	0.04275

cg19156483	2	Fign	-0.59788	0.042784
cg11383981	14	Lrtm1;Cacna2d3	1.52071	0.042794
cg22921453	18	Fem1c	-0.47156	0.042868
cg20014398	2	Pax6	1.88392	0.042884
cg04017326	19	Pik3ap1	0.656249	0.042972
cg25136495	6	Lpar5	1.790008	0.042989
cg20034190	11		1.947702	0.043058
cg21157465	4	Gm572	1.698363	0.043067
cg08218445	16	Mb21d2	1.865705	0.043107
cg27004639	12	Ttc7b	1.764511	0.043143
cg03681150	7	Mvp	0.400738	0.043168
cg23575668	16	Hic2	0.55703	0.043247
cg16473184	11	Rnf43	-0.45391	0.04329
cg08640766	2	Rbm12;Cpne1;Gm28036	0.587349	0.043305
cg00034468	8	Acta1;Galnt2l	1.780738	0.043336
cg07017063	13	Fst	-0.66921	0.043413
cg18712755	17	Prrc2a	0.620619	0.043469
cg07060006	9	Foxb1	-0.51146	0.043476
cg02836529	2	Neurod1;Cerkl	1.809015	0.043586
cg23388714	18	Mbd2	-0.39827	0.043626
cg12451099	2	Slc32a1	1.899538	0.043698
cg17633639	9	Dnajc13	1.248481	0.043712
cg23944405	2	Mpped2	1.751666	0.043739
cg12792180	11	Rbfox3	1.794542	0.044175
cg27537199	4		-0.72909	0.044206
cg08551218	17	Lrrc73	1.905974	0.044218
cg16775971	3	Cxxc4	2.138436	0.044219
cg19711800	4	Eri3	1.921025	0.044251
cg19148866	17	Six3	1.676823	0.044266
cg01219811	8	Nutf2	-0.40263	0.044294
cg24337081	2	Nup188	0.542791	0.044312
cg06084952	7	Fbxl19;Orai3	-0.55052	0.044374
cg05818894	2	Bdnf	-0.53653	0.044413
cg13034943	5	Ulk1	-0.60759	0.044597
cg23493031	12	Nkx2-1	2.005044	0.04461
cg13999774	1	Arpc2	-0.49668	0.04468
cg01614615	4	Prdm2	1.591133	0.044682
cg17132446	6	Hoxa4;Gm28308;Hoxa3	1.724845	0.044725
cg27397943	3	Zbtb10	-0.75874	0.044738

cg01488084	1	Map2	1.600741	0.04477
cg21824343	11	Wdr81	1.005485	0.0448
cg02007933	8	Galnt2l;Spata2l	-0.75865	0.044809
cg13870374	13	Tent4a;Nsun2	-0.9131	0.044873
cg01153660	15	Hoxc5;Hoxc6	-0.72061	0.044894
cg12432010	6	Iqsec3	1.661977	0.045003
cg20384231	7		1.321872	0.04501
cg13432391	9	Dnm2	1.738626	0.045045
cg14947478	2	Gdf5	1.949043	0.045079
ch.2.800013F	17	Birc6	1.671454	0.04518
cg14903727	19	Hnrnpul2;Gm50139	1.894697	0.045197
cg20917552	16		-0.6598	0.045405
cg07435294	19	Ovol1	1.917568	0.045505
cg25082847	14	Fezf2	-0.42839	0.045536
cg01572267	4	Elavl2	1.770203	0.045553
cg12440751	6	Kcna1	1.548934	0.045585
cg01579950	19	Nrxn2	-2.61939	0.045585
cg06030056	8	Sall1	-0.98984	0.045627
cg01272629	8		1.658747	0.045684
cg26264232	6	Hoxa2	-1.25055	0.04576
cg07268058	9	Isl2	1.879311	0.045765
cg05693527	6	Foxp1	-2.10183	0.045813
cg07197230	6	Cecr2	1.674893	0.045991
cg22992913	10	Agap2	1.639591	0.046011
cg13026772	9	Pknox2	1.763148	0.046024
cg08214610	X	Nkrf	1.936289	0.046082
cg09663101	16	Pi4ka;Snap29	-0.60183	0.046084
cg02690316	7	Fkrp;Strn4	1.709258	0.046112
cg27067760	12		1.486021	0.046118
cg14165096	1	Agap1	-0.98191	0.046122
cg22639787	2	Gnas	0.867703	0.046125
cg01923523	4	Lrrc47	1.86272	0.046188
cg10588135	11	Bcas3	2.16123	0.046225
cg14726968	4	Zcchc7	2.177397	0.046265
cg18001714	15	Ank	-0.53889	0.04639
cg06815950	5	Cux2	-0.54721	0.046421
cg10537807	11		1.812633	0.046459
cg19688796	1		-0.50181	0.046511
cg08448701	2	Pax1	1.968507	0.046571
cg20360212	11	Lif	2.001268	0.046585

cg21836062	X		1.764269	0.046605
cg06506831	6	Hoxa10	1.687729	0.046706
cg14325160	4	Rad23b	2.010611	0.046712
cg13023638	2	Kcnq2	1.828046	0.046737
cg20883310	10	Nuak1	1.786093	0.046868
cg06655377	8	Bbs2	1.821538	0.047158
cg15463484	7	Osbpl5	-0.63433	0.047198
cg01939220	6	Magi1	-0.56636	0.047214
cg05712938	13		1.205772	0.04725
cg12631713	4	Ajap1	-0.74788	0.04731
cg27604944	14	Dach1	2.109185	0.047368
cg20525279	8	Tenm3	2.044864	0.047382
cg06024079	11	Ftsj3;Psmc5	1.539153	0.04741
cg02184281	18	Smad7	1.547632	0.047526
cg15445628	2	Zmynd19	1.96616	0.047548
cg21129531	6	Snd1;Lrrc4	1.82637	0.047666
cg01106201	12	Arhgap5	1.147819	0.047688
cg27579480	8	Jund	1.535786	0.04769
cg12603946	4	Esrp1	2.105054	0.047726
cg14533651	6	Grin2b	-1.66124	0.047744
cg21884062	3	Mab21l1;Nbea	0.559718	0.047749
cg19445457	7	Olfr503	1.999738	0.047771
cg24805681	7	Mex3b	2.030146	0.047774
cg22143274	13		1.470173	0.04779
cg24536394	16	Son;Gart	1.809962	0.047817
cg17311022	16	Gm21987;Popdc2	2.379785	0.047826
cg09549726	7	Gabrb3	-1.97963	0.047874
cg14485809	19	Lbx1	1.443352	0.047875
cg06547959	18	Celf4	1.71646	0.047882
cg18236665	7	B9d2;Tgfb1	-1.49129	0.047997
cg19142001	2	Plcg1;Zhx3	-0.54763	0.048099
cg17122748	6	Plxna4	1.765868	0.048101
cg23665778	7	Dock1	1.612687	0.048115
cg10858898	11	Thra	1.838694	0.048147
cg06654549	13		2.047623	0.048156
cg14215077	2	Src	1.720079	0.048186
cg07620968	8	Mmp15	-0.5697	0.048349
cg11534937	5	Tnrc18	-1.07198	0.048363
cg01745428	2	Nol4l	-2.11842	0.048451
cg25811820	2	Plagl2;Pofut1	1.462741	0.048565
cg04645545	19	Fgf8	-1.98915	0.048614

cg12666411	3	Pogz	2.126774	0.048645
cg20876248	11	Eml6	-0.52908	0.048663
cg08549648	11	Tenm2	-0.48234	0.048698
cg27311392	X	Tsc22d3	-0.53191	0.048702
cg19236263	X	Trappc2;Ofd1	1.488897	0.048735
cg14940223	X	Armcx3	2.614923	0.048824
cg27359557	16	Lpp	0.777628	0.048832
cg20469625	15	Zfpm2	1.95247	0.04891
cg16626764	12	Ttc7b	1.850314	0.048945
cg15367487	7	Eif4g2	-1.7439	0.048945
cg01152019	2	Gm28230;Hoxd3;Hoxd4	1.353798	0.049026
cg18580296	12	Meox2	2.202355	0.049105
cg15364618	14	Ltb4r2	1.773441	0.049117
cg04998317	17		1.761079	0.049153
cg24980994	14	Zfp503	1.330146	0.049166
cg19918027	16	Kalrn	2.072572	0.049289
cg09735869	5	Rgs12	1.902714	0.04931
cg24591182	19	Plcb3	-1.46789	0.049312
cg03717442	17	Six3	-0.50267	0.049442
cg04118306	14	Nefm	1.421107	0.049629
cg13107760	2	Zfp804a	1.902453	0.049674
cg01746550	11		1.994309	0.049802
cg08611491	2	Jag1	1.450637	0.049849
cg03671597	11	Sez6	1.532499	0.049968

Supplementary Table S2. List of significant gene ontology pathways of three main categories, biological processes (BP), Cellular components (CC) and Molecular Functions (MF).

ID	category	description	count	zScore	*p*-value
GO:0006919	BP	GO_BP_MM_ACTIVATION_OF_CYSTEINE-TYPE_ENDOPEPTIDASE_ACTIVITY_INVOLVED_IN_APOPTOTIC_PROCESS	15	-0.2582	0.001647
GO:0042147	BP	GO_BP_MM_RETROGRADE_TRANSPORT_ENDOSOME_TO_GOLGI	7	0	0.002485
GO:0071549	BP	GO_BP_MM_CELLULAR_RESPONSE_TO_DEXAMETHASONE_STIMULUS	5	0	0.003545
GO:0030901	BP	GO_BP_MM_MIDBRAIN_DEVELOPMENT	22	1.788854	0.004751
GO:0000050	BP	GO_BP_MM_UREA_CYCLE	5	-1	0.005125
GO:0043406	BP	GO_BP_MM_POSITIVE_REGULATION_OF_MAP_KINASE_ACTIVITY	21	0	0.005739
GO:0016881	MF	GO_MF_MM_ACID-AMINO_ACID_LIGASE_ACTIVITY	38	-0.68599	0.006501
GO:0030139	CC	GO_CC_MM_ENDOCYTIC_VESICLE	11	-0.70711	0.007548
GO:0016311	BP	GO_BP_MM_DEPHOSPHORYLATION	50	0.617213	0.008449
GO:0008093	MF	GO_MF_MM_CYTOSKELETAL_ADAPTOR_ACTIVITY	7	0.377964	0.00884
GO:0001892	BP	GO_BP_MM_EMBRYONIC_PLACENTA_DEVELOPMENT	16	1	0.009087
GO:0072357	CC	GO_CC_MM_PTW_PP1_PHOSPHATASE_COMPLEX	5	0.447214	0.009362
GO:0008601	MF	GO_MF_MM_PROTEIN_PHOSPHATASE_TYPE_2A_REGULATOR_ACTIVITY	10	1.666667	0.010126
GO:0006470	BP	GO_BP_MM_PROTEIN_DEPHOSPHORYLATION	64	0.412082	0.010375
GO:0005614	CC	GO_CC_MM_INTERSTITIAL_MATRIX	7	-1.34164	0.010479

GO:0009378	MF	GO_MF_MM_FOUR-WAY_JUNCTION_HELICASE_ACTIVITY	7	-1.13389	0.011259
GO:0044255	BP	GO_BP_MM_CELLULAR_LIPID_METABOLIC_PROCESS	5	1.341641	0.011319
GO:0006520	BP	GO_BP_MM_CELLULAR_AMINO_ACID_METABOLIC_PROCESS	9	-1.63299	0.011831
GO:0034450	MF	GO_MF_MM_UBIQUITIN-UBIQUITIN_LIGASE_ACTIVITY	6	-1.63299	0.011858
GO:0030100	BP	GO_BP_MM_REGULATION_OF_ENDOCYTOSIS	9	0.377964	0.011977
GO:0016791	MF	GO_MF_MM_PHOSPHATASE_ACTIVITY	56	1.179536	0.012434
GO:0043525	BP	GO_BP_MM_POSITIVE_REGULATION_OF_NEURON_APOPTOTIC_PROCESS	19	1.697749	0.013272
GO:0032508	BP	GO_BP_MM_DNA_DUPLEX_UNWINDING	6	1.341641	0.014576
GO:0017137	MF	GO_MF_MM_RAB_GTPASE_BINDING	17	0.242536	0.015292
GO:0050729	BP	GO_BP_MM_POSITIVE_REGULATION_OF_INFLAMMATORY_RESPONSE	5	0	0.015485
GO:0006378	BP	GO_BP_MM_MRNA_POLYADENYLATION	11	2.110579	0.015515
GO:0055001	BP	GO_BP_MM_MUSCLE_CELL_DEVELOPMENT	5	-1	0.015805
GO:0035239	BP	GO_BP_MM_TUBE_MORPHOGENESIS	5	-1.41421	0.015841
GO:0035914	BP	GO_BP_MM_SKELETAL_MUSCLE_CELL_DIFFERENTIATION	8	0	0.015971
GO:0019722	BP	GO_BP_MM_CALCIUM-MEDIATED_SIGNALING	13	-0.57735	0.016049
GO:0032720	BP	GO_BP_MM_NEGATIVE_REGULATION_OF_TUMOR_NECROSIS_FACTOR_PRODUCTION	7	-0.8165	0.016719
GO:0006309	BP	GO_BP_MM_APOPTOTIC_DNA_FRAGMENTATION	5	0	0.017118

GO:0032147	BP	GO_BP_MM_ACTIVATION_OF_PROTEIN_KINASE_ACTIVITY	9	-1.41421	0.017602
GO:0016887	MF	GO_MF_MM_ATPASE_ACTIVITY	53	-0.14907	0.017808
GO:0004842	MF	GO_MF_MM_UBIQUITIN-PROTEIN_LIGASE_ACTIVITY	99	1.393746	0.019417
GO:0017069	MF	GO_MF_MM_SNRNA_BINDING	5	1	0.019589
GO:0030224	BP	GO_BP_MM_MONOCYTE_DIFFERENTIATION	9	1.414214	0.020505
GO:0043169	MF	GO_MF_MM_CATION_BINDING	6	-0.8165	0.020554
GO:0033280	BP	GO_BP_MM_RESPONSE_TO_VITAMIN_D	10	0.333333	0.021061
GO:0003148	BP	GO_BP_MM_OUTFLOW_TRACT_SEPTUM_MORPHOGENESIS	9	0.707107	0.021203
GO:0035413	BP	GO_BP_MM_POSITIVE_REGULATION_OF_CATENIN_IMPORT_INTO_NUCLEUS	5	-1.34164	0.021657
GO:0016829	MF	GO_MF_MM_LYASE_ACTIVITY	8	-0.70711	0.022199
GO:0008143	MF	GO_MF_MM_POLYA_RNA_BINDING	7	1.133893	0.022727
GO:0014911	BP	GO_BP_MM_POSITIVE_REGULATION_OF_SMOOTH_MUSCLE_CELL_MIGRATION	6	-2.23607	0.023303
GO:0034707	CC	GO_CC_MM_CHLORIDE_CHANNEL_COMPLEX	16	0.258199	0.02331
GO:0060216	BP	GO_BP_MM_DEFINITIVE_HEMOPOIESIS	10	0.632456	0.023373
GO:0008373	MF	GO_MF_MM_SIALYLTRANSFERASE_ACTIVITY	9	0.707107	0.023503
GO:0007018	BP	GO_BP_MM_MICROTUBULE-BASED_MOVEMENT	28	-0.40825	0.024463
GO:0045647	BP	GO_BP_MM_NEGATIVE_REGULATION_OF_ERYTHROCYTE_DIFFERENTIATION	5	1.341641	0.025107

GO:0003678	MF	GO_MF_MM_DNA_HELICASE_ACTIVITY	5	2	0.026183
GO:0031982	CC	GO_CC_MM_VESICLE	8	1.414214	0.026305
GO:0048037	MF	GO_MF_MM_COFACTOR_BINDING	7	0.816497	0.026396
GO:0017111	MF	GO_MF_MM_NUCLEOSIDE-TRIPHOSPHATASE_ACTIVITY	46	-0.52223	0.02643
GO:0043235	CC	GO_CC_MM_RECEPTOR_COMPLEX	11	1.414214	0.027581
GO:0010977	BP	GO_BP_MM_NEGATIVE_REGULATION_OF_NEURON_PROJECTION_DEVELOPMENT	16	1.069045	0.027728
GO:0071397	BP	GO_BP_MM_CELLULAR_RESPONSE_TO_CHOLESTEROL	5	2.236068	0.028226
GO:0030301	BP	GO_BP_MM_CHOLESTEROL_TRANSPORT	6	2	0.02862
GO:0046827	BP	GO_BP_MM_POSITIVE_REGULATION_OF_PROTEIN_EXPORT_FROM_NUCLEUS	5	1.732051	0.028647
GO:0010165	BP	GO_BP_MM_RESPONSE_TO_X-RAY	8	-1.13389	0.028792
GO:0033116	CC	GO_CC_MM_ENDOPLASMIC_RETICULUM-GOLGI_INTERMEDIATE_COMPARTMENT_MEMBRANE	10	-0.63246	0.02952
GO:0042787	BP	GO_BP_MM_PROTEIN_UBIQUITINATION_INVOLVED_IN_UBIQUITIN-DEPENDENT_PROTEIN_CATABOLIC_PROCESS	23	-0.22942	0.03014
GO:0030658	CC	GO_CC_MM_TRANSPORT_VESICLE_MEMBRANE	8	-1.34164	0.031128
GO:0051209	BP	GO_BP_MM_RELEASE_OF_SEQUESTERED_CALCIUM_ION_INTO_CYTOSOL	11	2.110579	0.031877
GO:0008277	BP	GO_BP_MM_REGULATION_OF_G-PROTEIN_COUPLED_RECEPTOR_PROTEIN_SIGNALING_PATHWAY	16	-0.90453	0.032528
GO:0060389	BP	GO_BP_MM_PATHWAY-RESTRICTED_SMAD_PROTEIN_PHOSPHORYLATION	9	0	0.033879

GO:0042162	MF	GO_MF_MM_TELOMERIC_DNA_BINDING	6	0.816497	0.034008
GO:0005892	CC	GO_CC_MM_ACETYLCHOLINE-GATED_CHANNEL_COMPLEX	7	-2.23607	0.034458
GO:0004629	MF	GO_MF_MM_PHOSPHOLIPASE_C_ACTIVITY	13	0.632456	0.035542
GO:0043116	BP	GO_BP_MM_NEGATIVE_REGULATION_OF_VASCULAR_PERMEABILITY	7	0.447214	0.03621
GO:0045995	BP	GO_BP_MM_REGULATION_OF_EMBRYONIC_DEVELOPMENT	7	-1.63299	0.03625
GO:0005160	MF	GO_MF_MM_TRANSFORMING_GROWTH_FACTOR_BETA_RECEPTOR_BINDING	5	1	0.03652
GO:0001965	MF	GO_MF_MM_G-PROTEIN_ALPHA-SUBUNIT_BINDING	7	-2.23607	0.037856
GO:0017017	MF	GO_MF_MM_MAP_KINASE_TYROSINE_SERINE_THREONINE_PHOSPHATASE_ACTIVITY	8	0.816497	0.03822
GO:0005179	MF	GO_MF_MM_HORMONE_ACTIVITY	20	1.147079	0.038265
GO:0005901	CC	GO_CC_MM_CAVEOLA	22	0.534522	0.038325
GO:0043204	CC	GO_CC_MM_PERIKARYON	27	-0.39223	0.038661
GO:0000062	MF	GO_MF_MM_FATTY-ACYL-COA_BINDING	5	1.341641	0.039161
GO:0005547	MF	GO_MF_MM_PHOSPHATIDYLINOSITOL-3_4_5-TRISPHOSPHATE_BINDING	12	0.904534	0.039464
GO:0006493	BP	GO_BP_MM_PROTEIN_O-LINKED_GLYCOSYLATION	6	0	0.040246
GO:0016331	BP	GO_BP_MM_MORPHOGENESIS_OF_EMBRYONIC_EPITHELIUM	10	0.707107	0.041461
GO:0002244	BP	GO_BP_MM_HEMOPOIETIC_PROGENITOR_CELL_DIFFERENTIATION	9	2.12132	0.04169
GO:0046325	BP	GO_BP_MM_NEGATIVE_REGULATION_OF_GLUCOSE_IMPORT	5	-1.34164	0.042011

GO:0009798	BP	GO_BP_MM_AXIS_SPECIFICATION	10	0	0.042077
GO:0019221	BP	GO_BP_MM_CYTOKINE-MEDIATED_SIGNALING_PATHWAY	18	0.258199	0.042382
GO:0004861	MF	GO_MF_MM_CYCLIN-DEPENDENT_PROTEIN_KINASE_INHIBITOR_ACTIVITY	6	1.732051	0.043699
GO:0004890	MF	GO_MF_MM_GABA-A_RECEPTOR_ACTIVITY	8	0.707107	0.044604
GO:0043508	BP	GO_BP_MM_NEGATIVE_REGULATION_OF_JUN_KINASE_ACTIVITY	6	-0.44721	0.044991
GO:0008158	MF	GO_MF_MM_HEDGEHOG_RECEPTOR_ACTIVITY	5	0.447214	0.04501
GO:0005865	CC	GO_CC_MM_STRIATED_MUSCLE_THIN_FILAMENT	5	0	0.046171
GO:0043401	BP	GO_BP_MM_STEROID_HORMONE_MEDIATED_SIGNALING_PATHWAY	37	1.616448	0.046869
GO:0009268	BP	GO_BP_MM_RESPONSE_TO_PH	6	0	0.048717
GO:0015813	BP	GO_BP_MM_L-GLUTAMATE_TRANSPORT	5	-1	0.048817
GO:0007422	BP	GO_BP_MM_PERIPHERAL_NERVOUS_SYSTEM_DEVELOPMENT	20	0.727607	0.049267
GO:0021895	BP	GO_BP_MM_CEREBRAL_CORTEX_NEURON_DIFFERENTIATION	6	0.447214	0.049628
GO:0007030	BP	GO_BP_MM_GOLGI_ORGANIZATION	13	0.83205	0.049714
GO:0042098	BP	GO_BP_MM_T_CELL_PROLIFERATION	6	0.816497	0.050112
GO:0046545	BP	GO_BP_MM_DEVELOPMENT_OF_PRIMARY_FEMALE_SEXUAL_CHARACTERISTICS	5	2	0.050233
GO:0007528	BP	GO_BP_MM_NEUROMUSCULAR_JUNCTION_DEVELOPMENT	14	-0.63246	0.050752
GO:0045861	BP	GO_BP_MM_NEGATIVE_REGULATION_OF_PROTEOLYSIS	6	0	0.05246
GO:0021631	BP	GO_BP_MM_OPTIC_NERVE_MORPHOGENESIS	5	-0.44721	0.053119

GO:0001574	BP	GO_BP_MM_GANGLIOSIDE_BIOSYNTHETIC_PROCESS	5	-2	0.053564
GO:0021846	BP	GO_BP_MM_CELL_PROLIFERATION_IN_FOREBRAIN	10	1.414214	0.053903
GO:0045786	BP	GO_BP_MM_NEGATIVE_REGULATION_OF_CELL_CYCLE	18	1.38675	0.054051
GO:0042523	BP	GO_BP_MM_POSITIVE_REGULATION_OF_TYROSINE_PHOSPHORYLATION_OF_STAT5_PROTEIN	5	0	0.054916
